# Measurement and modeling of particulate matter concentrations: Applying spatial analysis and regression techniques to assess air quality

**DOI:** 10.1016/j.mex.2017.09.006

**Published:** 2017-10-10

**Authors:** Seyed Ali Sajjadi, Ghasem Zolfaghari, Hamed Adab, Ahmad Allahabadi, Mehri Delsouz

**Affiliations:** a Department of Environmental Health Engineering, Faculty of Health, Gonabad University of Medical Sciences, Gonabad, Iran; b Department of Environmental Sciences and Engineering, Faculty of Geography and Environmental Sciences, Hakim Sabzevari University, Sabzevar, Iran; c Department of Climatology and ​Geomorphology, Faculty of Geography and Environmental Sciences, Hakim Sabzevari University, Sabzevar, Iran; d Department of Environmental Health Engineering, Faculty of Health, Sabzevar University of Medical Sciences, Sabzevar, Iran

**Keywords:** PM_2.5_ and PM_10_, Monitoring, Spatial modeling

## Abstract

This paper presented the levels of PM_2.5_ and PM_10_ in different stations at the city of Sabzevar, Iran. Furthermore, this study was an attempt to evaluate spatial interpolation methods for determining the PM_2.5_ and PM_10_ concentrations in the city of Sabzevar. Particulate matters were measured by Haz-Dust EPAM at 48 stations. Then, four interpolating models, including Radial Basis Functions (RBF), Inverse Distance Weighting (IDW), Ordinary Kriging (OK), and Universal Kriging (UK) were used to investigate the status of air pollution in the city. Root Mean Square Error (RMSE), Mean Absolute Error (MAE) and Mean Absolute Percentage Error (MAPE) were employed to compare the four models. The results showed that the PM_2.5_ concentrations in the stations were between 10 and 500 μg/m^3^. Furthermore, the PM_10_ concentrations for all of 48 stations ranged from 20 to 1500 μg/m^3^. The concentrations obtained for the period of nine months were greater than the standard limits. There was difference in the values of MAPE, RMSE, MBE, and MAE. The results indicated that the MAPE in IDW method was lower than other methods: (41.05 for PM_2.5_ and 25.89 for PM_10_). The best interpolation method for the particulate matter (PM_2.5_ and PM_10_) seemed to be IDW method.

• The PM_10_ and PM_2.5_ concentration measurements were performed in the period of warm and risky in terms of particulate matter at 2016.
• Concentrations of PM_2.5_ and PM_10_ were measured by a monitoring device, environmental dust model Haz-Dust EPAM 5000.
• Interpolation is used to convert data from observation points to continuous fields to compare spatial patterns sampled by these measurements with spatial patterns of other spatial entities.

## Graphical abstract

**Figure:**
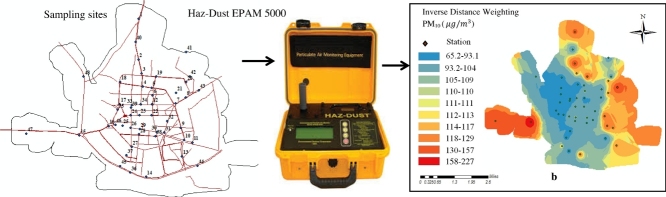

## Methods details

### Description of area

**Fig. 1:**
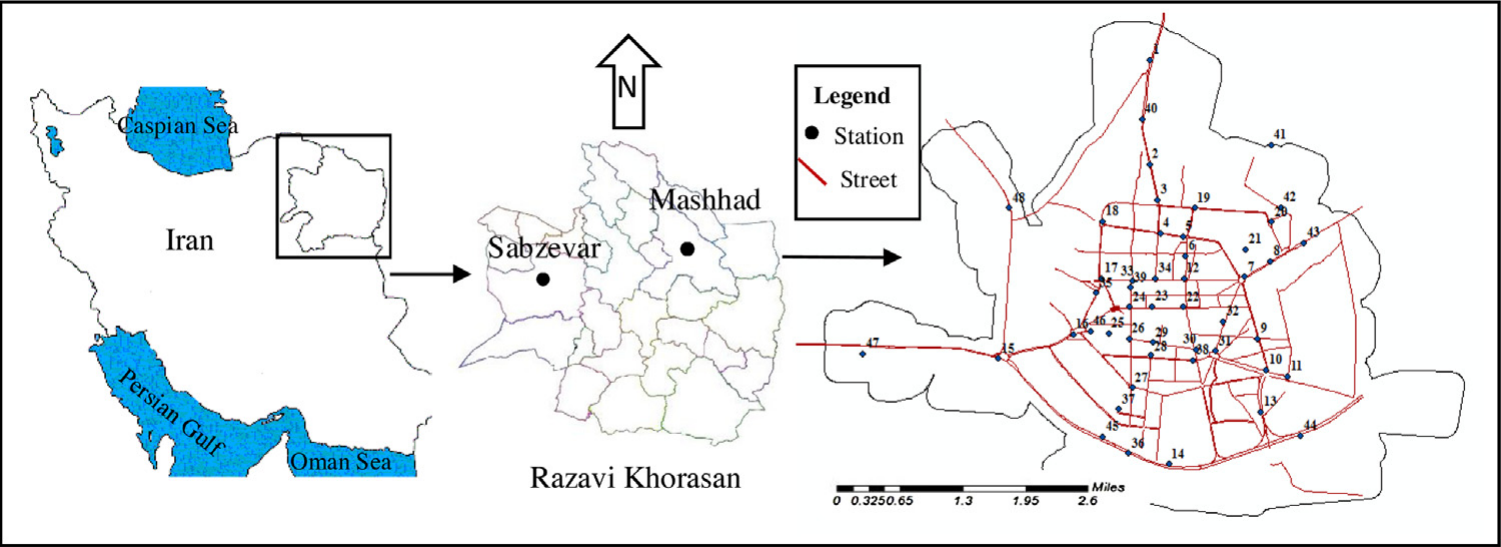
Geographical situation of areas in which the samples were collected, Sabzevar city in Razavi Khorasan province, Iran.

Sabzevar is located in Razavi Khorasan province, Iran, extending from the east longitude of 56° 04′ to 58° 15′ and northern latitude of 35° 30′ to 36° 58′ [1]. This city is located between Neyshabour from east, Esfarayen from north, Bardaskan from south, and Shahrood from west. This is a fairly large region (23 km^2^) with a population of 231,557 [2]. It is situated at elevation 977 m above sea level. It operates on the IRDT time zone, following the same time zone as Mashhad, the capital of Razavi Khorasan province (Fig. 1).

### Sample collection

The PM_10_ and PM_2.5_ concentration measurements were performed in the period of warm and risky in terms of particulate matter at 2016. A total of 246 samples were collected during April 2016–December 2016 for nine month for each PM (PM_2.5_ and PM_10_) from various positions including residential, traffic light, traffic junction, and commercial sites. Concentrations of PM_2.5_ and PM_10_ were measured by a monitoring device, environmental dust model Haz-Dust EPAM 5000. The Haz-Dust EPAM 5000 is a high sensitive real-time particulate monitor designed for ambient environmental and indoor air quality applications. This unit combines traditional filter techniques with real-time monitoring methods. These combined techniques can overcome the limitations of all other aerosol monitoring products. The Haz-Dust EPAM-5000 uses the principle of near-forward light scattering of an infrared radiation to immediately and continuously measure the concentration of airborne dust particles in mg/m^3^. Sampling stations were selected according to the study manual for the European Study of Cohorts for Air Pollution Effects [3]. The monitoring sites were located at 25 m of a traffic light or a traffic junction, with a sampling height at least 1.5 m above the ground. Furthermore, monitoring sites were not located within 25 m of locations where smokers are allowed to smoke and/or gather (for example close to the entrance of restaurants, hospitals, schools or other public buildings), because smokers usually congregate close to the entrance of these buildings. Instrument calibration was done according to the procedure adopted by Environmental Devices Corporation. A GPS (Global Positioning System) instrument with UTM system (Model eTrex Vista) was used for geographical positioning (X: longitude and Y: latitude). The names of the sampling sites and their related geographical coordinates in Sabzevar are listed in Table 1. Concentrations reported in Table 1 were measured in a 9-month study period.

**Table 1:** The names of thesampling sites, their related geographical coordinates, and particulate matter concentrations (μg/m^3^).^a^

Station	X^b^	Y^b^	No.	Urban fabric	PM_2.5_ (mean ± S.E)	Range	PM_10_ (mean ± S.E)	Range	PM_2.5_/PM_10_
1- Vasee Hospital	560739	4011590	4	Boulevard	67.50 ± 27.50	40–150	142.50 ± 52.50	90–300	0.47
2- Towhid	560726	4009962	4	Town	87.50 ± 37.50	50–200	185.00 ± 105.00	80–500	0.47
3- Emdad	560850	4009410	9	Cross	64.44 ± 24.15	10–250	128.80 ± 35.17	50–400	0.50
4- Fahmideh	560894	4008890	4	Square	41.25 ± 11.25	30–75	55.00 ± 15.00	40–100	0.75
5- Laleh	561250	4008838	4	Square	62.50 ± 22.50	40–130	82.50 ± 22.50	60–150	0.75
6- Motahari	561295	4008542	4	Avenue	62.50 ± 12.50	50–100	122.50 ± 2.50	120–130	0.51
7- Shariati	562215	4008214	6	Square	43.33 ± 11.45	30–100	76.66 ± 16.86	40–150	0.56
8- Sherkati	562611	4008456	4	Oil station	82.50 ± 22.50	60–150	122.50 ± 42.50	80–250	0.67
9- Ghand va Shekar	562412	4007252	4	Cross	87.50 ± 2.50	80–90	152.50 ± 32.50	120–250	0.57
10- Emam Hosain	562552	4006760	5	Square	78.00 ± 30.56	40–200	152.00 ± 62.08	80–400	0.51
11-Mosalla	562885	4006661	4	Square	47.50 ± 17.50	30–100	122.50 ± 42.50	80–250	0.38
12- Saheb −al- Zaman	561263	4008186	4	Square	55.00 ± 15.00	40–100	82.50 ± 22.50	60–150	0.66
13- Emam Hosain	562462	4006115	4	Boulevard	55.00 ± 15.00	40–100	68.75 ± 18.75	50–125	0.80
14- Beyhaq	561029	4005296	8	Oil station	100 ± 36.30	40–350	131.25 ± 39.02	60–400	0.76
15- Sarbedaran	558374	4006943	13	Square	107.69 ± 34.99	20–500	234.62 ± 107.56	30–1500	0.42
16- Shahid Beheshti	559539	4007320	6	Square	50.00 ± 10.95	20–100	60.83 ± 13.44	30–125	0.82
17- Enghelab	559987	4008194	7	Square	51.42 ± 15.64	20–120	80.00 ± 20.23	30–160	0.64
18- Kushk	560002	4009080	6	Square	43.33 ± 12.01	20–100	78.33 ± 18.87	30–150	0.55
19- Razi	561432	4009287	4	Avenue	40.00 ± 20.00	20–100	162.50 ± 112.50	50–500	0.24
20- Razi	562627	4009071	4	Square	47.50 ± 17.50	30–100	122.50 ± 42.50	80–250	0.38
21- Azad University	562234	4008640	4	Street	47.50 ± 17.50	30–100	82.50 ± 22.50	60–150	0.57
22- Dadgostari	561260	4007744	7	Cross	55.71 ± 15.86	30–150	92.85 ± 18.47	60–200	0.60
23- Hakim	560765	4007752	4	Square	40.00 ± 20.00	20–100	112.5 ± 62.50	50–300	0.35
24- Kushk	560414	4007744	5	Cross	44.00 ± 14.35	20–100	62.00 ± 22.22	30–150	0.70
25- Bazar-e-Ruz	560094	4007333	4	Square	55.00 ± 15.00	40–100	82.50 ± 22.50	60–150	0.66
26- Darvaz-e-Araq	560411	4007246	4	Square	55.00 ± 15.00	40–100	115.00 ± 5.00	110–130	0.47
27- Resalat	560460	4006490	4	Avenue	47.50 ± 17.50	30–100	82.50 ± 22.50	60–150	0.57
28- Asrar	560754	4006995	8	Square	51.00 ± 11.54	10–100	90.00 ± 21.21	20–200	0.56
29- Beyhaq	560776	4007194	10	Cross	75.40 ± 15.21	30–200	124.00 ± 31.83	50–400	0.60
30- Post va Telegraph	561452	4007085	9	Square	66.00 ± 17.55	20–200	114.44 ± 32.23	30–350	0.57
31- Kargar	561754	4007056	4	Square	62.50 ± 12.50	50–100	82.50 ± 22.50	60–150	0.75
32- Abumoslem	561876	4007518	4	Avenue	27.50 ± 7.50	20–50	82.50 ± 22.50	60–150	0.33
33- Jalal al Ahmad	560471	4008148	4	Square	27.50 ± 7.50	20–50	82.50 ± 22.50	60–150	0.33
34- Tabas	560825	4008187	11	Square	52.72 ± 7.98	30–100	98.18 ± 16.22	60–250	0.53
35- Hoveyze	559901	4007964	4	Avenue	27.50 ± 7.50	20–50	82.50 ± 22.50	60–150	0.33
36- Mashhad	560399	4005465	4	Highway	27.50 ± 7.50	20–50	82.50 ± 22.50	60–150	0.33
37- Resalat	560248	4006154	4	Oil station	27.50 ± 7.50	20–50	82.50 ± 22.50	60–150	0.33
38- Modares	561406	4006913	4	Boulevard	27.50 ± 7.50	20–50	82.50 ± 22.50	60–150	0.33
39- Emam Ali	560440	4008047	4	Oil station	27.50 ± 7.50	20–50	82.50 ± 22.50	60–150	0.33
40- University	560610	4010673	4	Boulevard	27.50 ± 7.50	20–50	82.50 ± 22.50	60–150	0.33
41- North Beltway	562625	4010264	4	Boulevard	27.50 ± 7.50	20–50	82.50 ± 22.50	60–150	0.33
42- Azad University	562772	4009294	4	Street	27.50 ± 7.50	20–50	82.50 ± 22.50	60–150	0.33
43- Ghuchan	563129	4008739	4	Road (first of)	100.00 ± 40.00	60–220	200.00 ± 100.00	100–500	0.50
44- Mashhad	563085	4005747	4	Road (first of)	27.50 ± 7.50	20–50	122.50 ± 42.50	80–250	0.22
45- Vegetable Field	560004	4005722	4	High Way	67.50 ± 27.50	40–150	122.50 ± 42.50	80–250	0.55
46- Beyhaq	559805	4007366	4	Avenue	27.50 ± 7.50	20–50	82.50 ± 22.50	60–150	0.33
47- Police	556256	4007013	4	Road (first of)	47.50 ± 17.50	30–100	162.50 ± 112.50	50–500	0.29
48- Esfarayen	558544	4009290	4	Road (first of)	67.50 ± 27.50	40–150	102.50 ± 32.50	150–200	0.65

a Sampling stations were selected according to study manual for the European Study of Cohorts for Air Pollution Effects, ESCAPE [3]. Concentrations reported in table are in a 9-month study period.

b X: longitude and Y: latitude.

### Interpolation methods

Interpolation is a procedure to predict the value of attributes at non-sampled sites from measurements made at point locations within the same area. Interpolation is used to convert data from observation points to continuous fields to compare spatial patterns sampled by these measurements with spatial patterns of other spatial entities. The rationale behind spatial interpolation is the very common observation that, on average, values at points close together in space are more likely to be similar than points further apart. Among spatial interpolation methods, one can find RBF, IDW, and Kriging techniques [4]. RBF methods predict values that can vary above the maximum or below the minimum of the measured values. For all RBF methods, a parameter controls the smoothness of the resulting surface. By using radial basis functions, dealing with higher dimensional problems in a similar way as is possible dealing with two- and three-dimensional problems. Splines (RBF) are interpolators fitting a function for sampled points. The algorithm uses a linear combination of *n* functions, one for each known point as demonstrated in the following equation:

(1) $$\hat{Z}(S_{0})=\overset{n}{\underset{i=1}{\sum }}\omega_{i}\varphi (S_{i}-S_{0})+\omega_{n+1}$$

where *φ(r)* represent the interpolation function, *S_i_* − *S*_0_ the Euclidean distance *r* between an unknown point *S*_0_ and an observed one *S*_i_, while *ω*_i_, with *i = 1*, *2*,*…*,*n + 1*, are weight. Weights are assigned according to the distance of known points, under the constraint that, in their locations, the function must give the measured value. Radial Basis Functions (RBF) is a family of five deterministic exact interpolation techniques [5], [6], [7] as below:

Thin-plate Spline function:

(2) *φ(r) = (σ.r)^2^ ln (σ.r)*

Multi-quadric function:

(3) $$\varphi (r){\left[r^{2}+\sigma^{2}\right]}^{\frac{1}{2}}$$

Inverse Multi-quadric function:

(4) $$\varphi (r)={\left[r^{2}+c^{2}\right]}^{-\frac{1}{2}}$$

Completely regularized Spline function:

(5) $$\varphi (r)=-\overset{\infty }{\underset{n=1}{\sum }}\frac{{\left(-1\right)}^{n}.r^{2n}}{n!n}=ln{\left[\frac{\sigma .r}{2}\right]}^{2}+E_{1}{\left[\frac{\sigma .r}{2}\right]}^{2}+C_{E}$$

Spline with tension function:

(6) $$\varphi (r)=ln\left[\frac{\sigma .r}{2}\right]+K_{0}{\left(\sigma .r\right)}^{2}+C_{E}$$

where *r* = distance between the point and the sample, *σ* = tension factor, *E*_1_ = exponential integral function, *C*_E_ = constant of Eulero (0.577215), and *K*_0_ = modified Bessel function.

Splines functions are slightly different, each one has a different smoothing parameter depending on the σ parameter. In every method, the higher the value of *σ*, the higher the gradualness of the variation, except for the inverse multi-quadric where the opposite condition is true. The regularized Spline creates a smooth, gradually changing surface. The regularizing parameter is in fact employed to achieve a smoother solution: e.g. a small value results in a close approximation of the data, while a large one results in a smoother solution [8].

IDW interpolates all values of the points within the sample range as averaging tool and gives better interpolation estimates when the minimum and maximum values of the surface are represented by sample data points. The concentration of point will have heavier weight if it is proximal to the required point and vice versa. Here, weight is an inverse function of the distance, as demonstrated in the following equation:

(7) $$\begin{array}{ccc} Z_{j}=\frac{\sum_{i=1}^{n}W_{i}Z_{i}}{\sum_{i=1}^{n}W_{i}} & and & W_{i}=\frac{1}{d_{ji}^{p}} \end{array}$$

where *Z*_j_ is the concentration at the *j*^th^ point, *W*_i_ is the weight of observed *i*^th^ point, *d*_ji_ is the distance from the *i*^th^ point to the *j*^th^ point, *p* is the power and *n* is total number of points [9]. The IDW functions used in this study are power 1 and power 2.

Recognizing that the spatial variation of any continuous attribute is often too irregular to be modelled by a simple, smooth mathematical function, Kriging is a wide family of interpolation methods using geostatistics. Geostatistical methods for interpolation rely on the assumption of spatial autocorrelation. This suggests that the distance and direction between sample points are the major factors governing the estimated values at unknown points. With spatial autocorrelation, the function used for data fitting provides better estimates. Kriging methods include Ordinary, Simple, Universal, Indicator, Probability, Disjunctive Kriging, and Co-Kriging, which all rely on the concept of autocorrelation. The Ordinary Kriging Eq. (8) represents a weighted sum of the data, which is:

(8) $$Z\left(S_{0}\right)=\overset{N}{\underset{i=1}{\sum }}\lambda_{i}Z\left(S_{i}\right)$$

where $Z\left(S_{0}\right)$ is the predicted value at location *S*_0_, N is the number of samples used for the estimation, $Z\left(S_{\text{i}}\right)$ is the measured value at location i, *λ*_i_ is the unknown weight for the measured value at *i*^th^ location determined using the fitted variogram [4]. The aim of the UK method is to predict Z(x) at a non-sampled area as well. It splits the random function into a linear combination of deterministic functions, the smoothly varying and non-stationary trend, that is also called a drift μ(x) ϵ R, and a random component Y(x): = Z(x) − μ(x), representing the residual random function [10]. OK assumes a stationary, i.e. constant mean of the underlying real-valued random function Z(x). But in reality, the mean value varies, it is not often constant across the entire study area and the variable seems to be non-stationary. A non-stationary regionalized variable can be considered with two components [11]; drift (average or expected value of the regionalized variable) and a residual (difference between the actual measurements and the drift). The method of UK assumes that the mean m(x) has a functional dependence on the spatial location and can be approximated by a model with the equation [12]:

(9) $$\mu (x)=\overset{k}{\underset{l=1}{\sum }}a_{l}f_{l}(x)$$

where *a*_l_ is *l*_th_ coefficient to be estimated from the data, *f*_l_ is *l*_th_ basic function of spatial coordinates that describes the drift, and *k* is the number of functions used in modeling the drift. The OK and UK functions used in this study are including: stable, hole effect, J-Bessel, and Gaussian.

### Model evaluation

After the interpolation, each spatiotemporal point in the check data set would have both the original PM_2.5_ and PM_10_ measurements and an estimated value. Then we conducted four accuracy assessments to compare the original and estimated PM_2.5_ and PM_10_ values, including MAPE (Mean Absolute Percentage Error), RMSE (Root Mean Square Error), MBE (Mean Bias Error), and MAE (Mean Absolute Error). In all equations, *N* is the number of observations, *I*_i_s are the interpolated values, and *O*_i_s are the original values [13]. The accuracy assessments are defined as follows (Eqs. (10)–(13)):

(10) $$MAPE=\frac{100}{N}\times \overset{N}{\underset{i-1}{\sum }}\left|\frac{I_{i}-O_{i}}{O_{i}}\right|$$

The MAPE, also known as Mean Absolute Percentage Deviation (MAPD), is a measure of prediction accuracy of a forecasting method in statistics, for example in trend estimation. It usually expresses accuracy as a percentage.

(11) $$RMSE=\sqrt{\frac{\sum_{i=1}^{N}{\left(I_{i}-O_{i}\right)}^{2}}{N}}$$

The RMSE has been used as a standard statistical metric to measure model performance in meteorology, air quality, and climate research studies.

(12) $$MBE=\frac{\sum_{i=1}^{N}\left(I_{i}-O_{i}\right)}{N}$$

The MBE statistical indicator is also commonly used in comparing the models of predictions. Low values of MBE are desirable, but overestimation of an individual data element will cancel underestimation in a separate observation.

(13) $$MAE=\frac{\sum_{i=1}^{N}\left|I_{i}-O_{i}\right|}{N}$$

The MAE is another useful measure widely used in model evaluations. The MAE is a quantity used to measure how close forecasts or predictions are to the eventual outcomes.

### Statistical analysis

Statistical description of data (PM_2.5_ and PM_10_) for interpolation techniques was performed by XLSTAT. The spatial structure analyzing data and zooning by interpolation methods include: Ordinary Kriging, Universal Kriging, Inverse Distance Weighting, and Radial Basis Functions done by Arc GIS software version 10.3. Finally, the best interpolation method was chosen according to the values of each error algorithm. It should be noted that in case of normal data, interpolation methods will have the best results. In case of non-normal data distribution, we can do normalization by “Cox-box”. The statistical analysis for geographical comparisons was done by the SPSS software (Chicago, IL, USA, Version 16.0). The data were tested for normality using a Shapiro–Wilk test. The data were not normally distributed. We used non-parametric procedures, and Kruskal–Wallis test followed by a Mann–Whitney *U* test. A multiple regressions was calculated between particulate matter and independent meteorological parameters. The Spearman correlation test was used to examine the relationship between PM _2.5_ and PM_10_ particulates as well as between particulate matter and meteorological parameters.

### Particulate matter standards

**Fig. 2:**
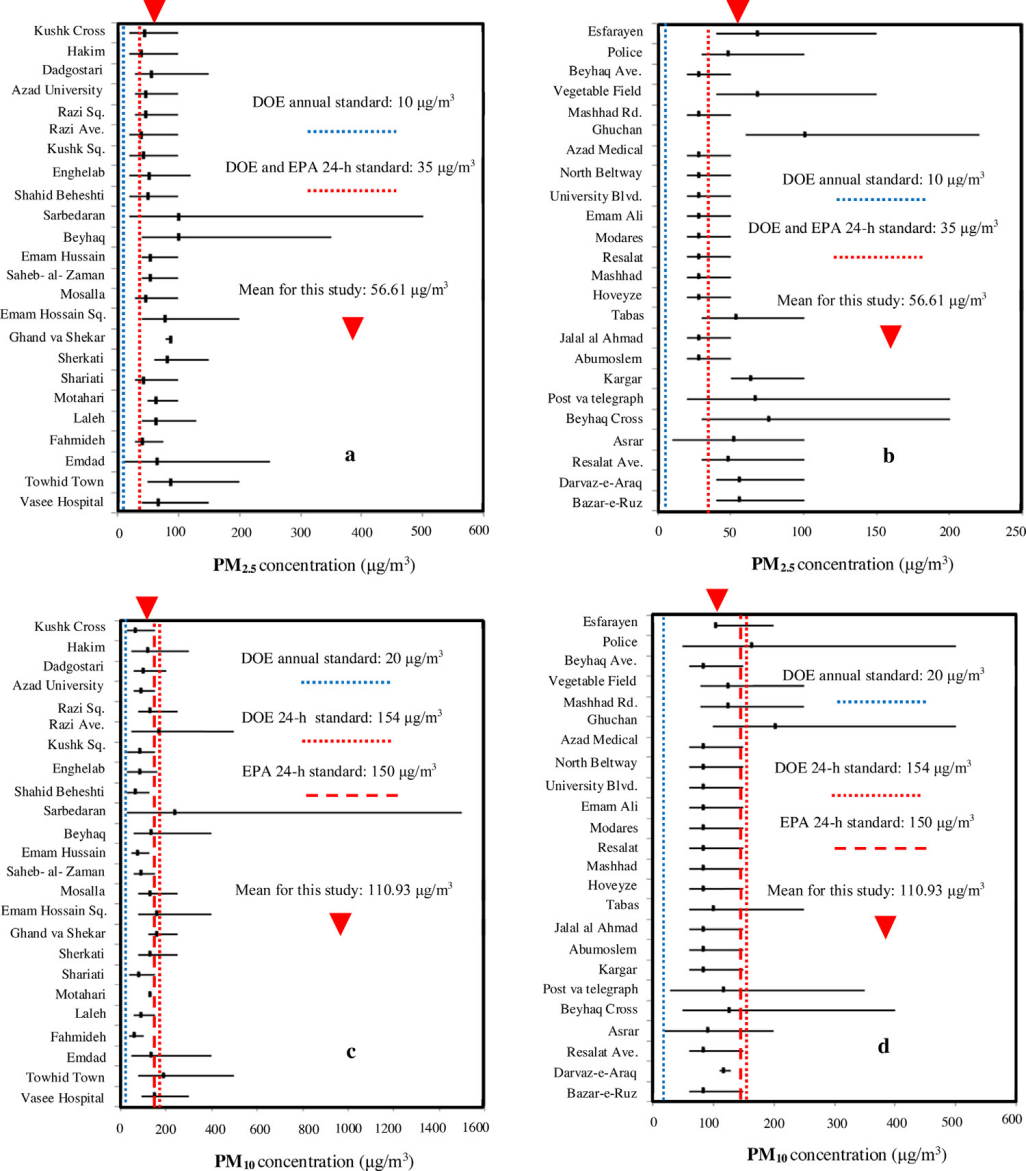
Levels of PM concentration (μg/m^3^) in urban areas of Sabzevar. (a and b) concentration of PM_2.5_. (c and d) concentration of PM_10_. The horizontal line is the range, and tick on the horizontal line is the mean. The red triangle identifies the mean value for the present study. The vertical dashed and dotted lines are the DOE (Department of Environment, Iran) and EPA (Environmental Protection Agency, USA) standards.

Based on standard of Iran Department of Environment (DOE) in 2014 which considered the annual maximum concentration for PM_2.5_ as 10 μg/m^3^, the values obtained for the period of nine months were greater than the standard limit; but based on Environmental Protection Agency (EPA) standard in 2012 and standard of DOE, which announced the maximum concentrations of 24-h for PM_2.5_ as 35 μg/m^3^, the results were greater than standard in 75% of cases (Fig. 2). Regarding the standard of DOE in 2014, which announced the annual maximum concentration for PM_10_ as 20 μg/m^3^, the values obtained for the period of nine months were greater than the standard limit, but compared with DOE 24-h for PM_10_ as 154 μg/m^3^, the values were less than the strategy values in 89.58% of the cases. Furthermore, in comparison with EPA 24-h for PM_10_ as 150 μg/m^3^, the values were less than the strategy values in 87.5% of the cases. In similar studies, the great amount of aerosol particles has been confirmed in other cities of Iran [14]. The ratio of PM_2.5_/PM_10_ showed variability from 0.22 (Mashhad Road station) to 0.82 (Shahid Beheshti Square). It indicates that coarse particles (greater than 2.5 μm) make up the majority of aerosol (Table 1). In similar studies by USEPA, the annual mean PM_2.5_/PM_10_ ratios measured in urban and semi-rural areas were between 0.3 and 0.7 [15]. The average PM_2.5_/PM_10_ ratio during the sampling period was 0.50 compared to the range from 0.15 to 0.25, reported by EPA [16]. The percentage ratio of the mean concentrations of PM_10_/PM_2.5_/PM_1.0_ in Tehran was found to be approximately as 7: 2: 1 [17].

### Monitoring

**Fig. 3:**
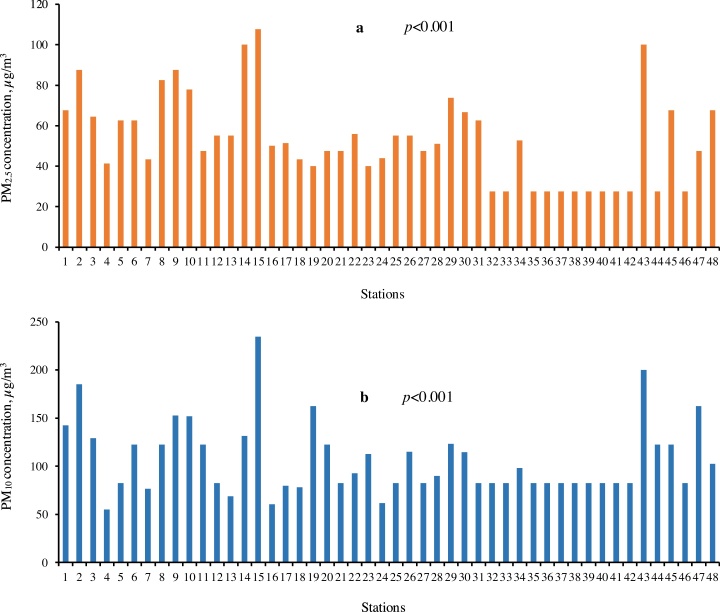
Statistical differences among the studied stations in Sabzevar. (a) The mean concentration of PM_2.5_ and (b) The mean concentration of PM_10_. Concentrations reported in the Figure are in 9-month study period. 1: Vasee Hospital, 2: Towhid, 3: Emdad, 4: Fahmideh, 5: Laleh, 6: Motahari, 7: Shariati, 8: Sherkati, 9: Ghand va Shekar, 10: Emam Hosain Square, 11: Mosalla, 12: Saheb- al- Zaman, 13: Emam Hosain Boulevard, 14: Beyhaq Oil station, 15: Sarbedaran, 16: Shahid Beheshti, 17: Enghelab, 18: Kushk, 19: Razi Avenue, 20: Razi Square, 21: Azad University, 22: Dadgostari, 23: Hakim, 24: Kushk, 25: Bazar-e-Ruz, 26: Darvaz-e-Araq, 27: Resalat, 28: Asrar, 29: Beyhaq Cross, 30: Post va Telegraph, 31: Kargar, 32: Abumoslem, 33: Jalal al Ahmad, 34: Tabas, 35: Hoveyze, 36: Mashhad, 37: Resalat, 38: Modares, 39: Emam Ali, 40: University Boulevard, 41: North Beltway, 42: Azad Medical University, 43: Ghuchan, 44: Mashhad, 45: Vegetable Field, 46: Beyhaq, 47: Police, Sabzevar- Tehran, and 48: Esfarayen.

The mean concentrations of particulate matter in 48 sampling stations during the ninth month study period for present research are shown in Table 1 and Fig. 3. The results showed that the PM_2.5_ concentrations in the stations were between 10 and 500 μg/m^3^, and there was a significant effect among the stations in relation to PM_2.5_ concentrations (*p <* 0.001). The highest PM_2.5_ concentration was in Sarbedaran Square (107.69 μg/m^3^). Fahmideh Square and Hakim Square had intermediate values (41.25 and 40.00 μg/m^3^, respectively), and the lowest concentrations of PM_2.5_ were in Abumoslem Avenue, Jalal al Ahmad Square, Hoveyze Avenue, Mashhad Highway station, Resalat oil station, Modares Boulevard, Emam Ali oil station, University Boulevard, North Beltway, Azad Medical University, Mashhad Road station, and Beyhaq Avenue station (27.50 μg/m^3^). The PM_10_ concentrations for all of 48 stations ranged from 20 to 1500 μg/m^3^. We found a significant difference in PM_10_ concentrations among the stations (*p <* 0.001) with highest concentrations in Sarbedaran Square (234.62 μg/m^3^) followed by Ghuchan Road, Towhid Town, and Police (Sabzevar-Tehran) station. Enghelab Square had intermediate values (80 μg/m^3^), followed by Kushk Square, Shariati Square, Emam Hosain Boulevard, Kushk Cross, Shahid Beheshti Square, whereas Fahmideh Square contained the least amount of PM_10_ (55 μg/m^3^). The main reason for the high concentration of particulate matter in the Ghuchan Road station was the occurrence of strong wind entered from the east Sabzevar that it has significant impact on the air quality of the city. This station has a marginal position and is devoid of vegetation. Towhid station also has a high concentration. The station is located in the north Sabzevar. The lack of vegetation and construction activities could be due to high concentrations. The pollution in Sarbedaran station is caused by high traffic of heavy vehicles in the Mashhad highway located in this region. Furthermore, the main reason for the pollution in Police station, Sabzevar- Tehran is industrial activities in this region. In the south of Beyhaq Oil station, agricultural activities take place that can be a source of particulate matter.

In urban agglomerations, there are different and various point and line sources of particles. Whereas industrial activities such as domestic heating with coal or oil are seen as point sources, emission of particles from motorized traffic occurs mainly along the roads to be realized a line source. Emissions by motorized vehicles do not only include exhaust particles, but also involve abrasion products from tyres, brakes, clutches, and the road’s surface. Furthermore, particles are emitted by re-suspension of previously deposited particles by vehicle-induced turbulence. Besides the local emissions, particle concentrations in cities are also influenced by advection due to particle transport from rural surroundings or long-range, often trans-boundary transport. Moreover, the local winds seem to contribute to pollution in Sabzevar. In a study in India, it was shown that presence of dust and great traffic affected the amount of particles by 42.6% and 3.36% [18]. In Srilanka, the average of PM_2.5_ concentration varied from 18 to 83 μg/m^3^ in outdoor [19]. In a study in 6 municipal zones of Chile, the amounts of particles were greater in the central regions of the cities [20]. As shown in Table 1 and Fig. 3, the concentration of PM_10_ is much higher than the PM_2.5_. It seems that PM_10_ as pollutant is responsible for the pollution of Sabzevar. According to research conducted in USA [21], PM measurement results showed that highway and marginal urban areas by improving traffic bottleneck, the amount of particulate matter is reduced to 41 percent. The studies of on the mass PM_2.5_ and PM_1_ effects in Helsinki urban air pollution showed that PM_2.5_ is the most effective PM index for air pollution which is most significantly associated with respiratory and Cardiovascular disease [22]. Measurements of PM_10_ and PM_2.5_ in urban area of Nanjing, China showed that more than 70% of total suspended particles are of a size that they are deposited in the respiratory tract below trachea, whereas about 22% of the mass is respirable and will reach the alveoli [23].

### Spatial modeling

**Fig. 4:**
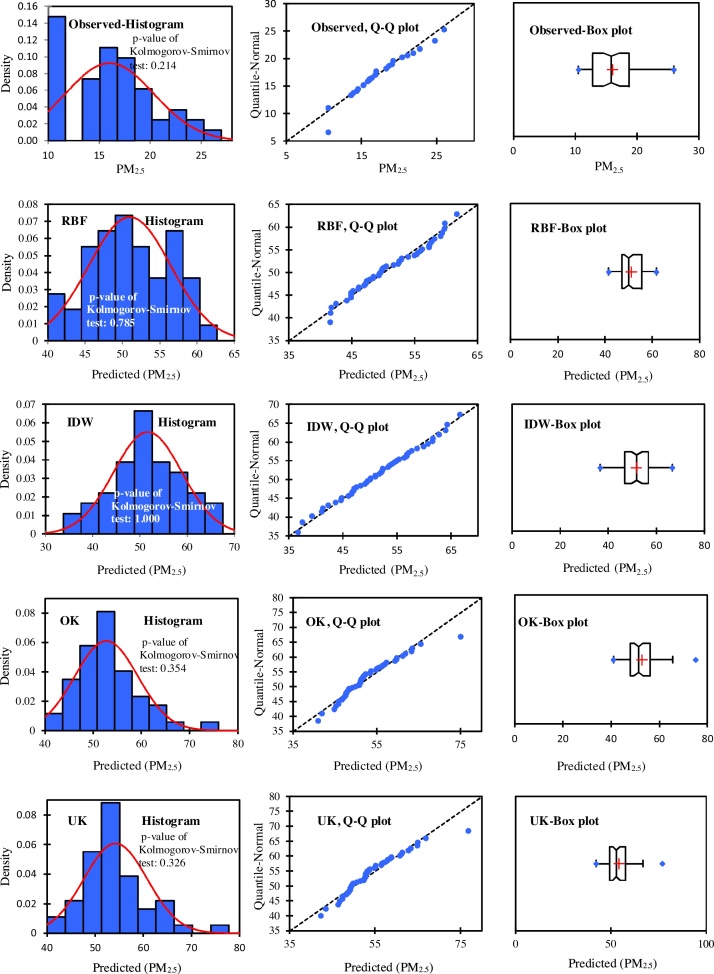
Comparison among the distributions of observed data, the predicted data using IDW, RBF, OK (stable function), and UK (stable function) for PM_2.5_. The figures were drawn by XLSTAT software.

**Fig. 5:**
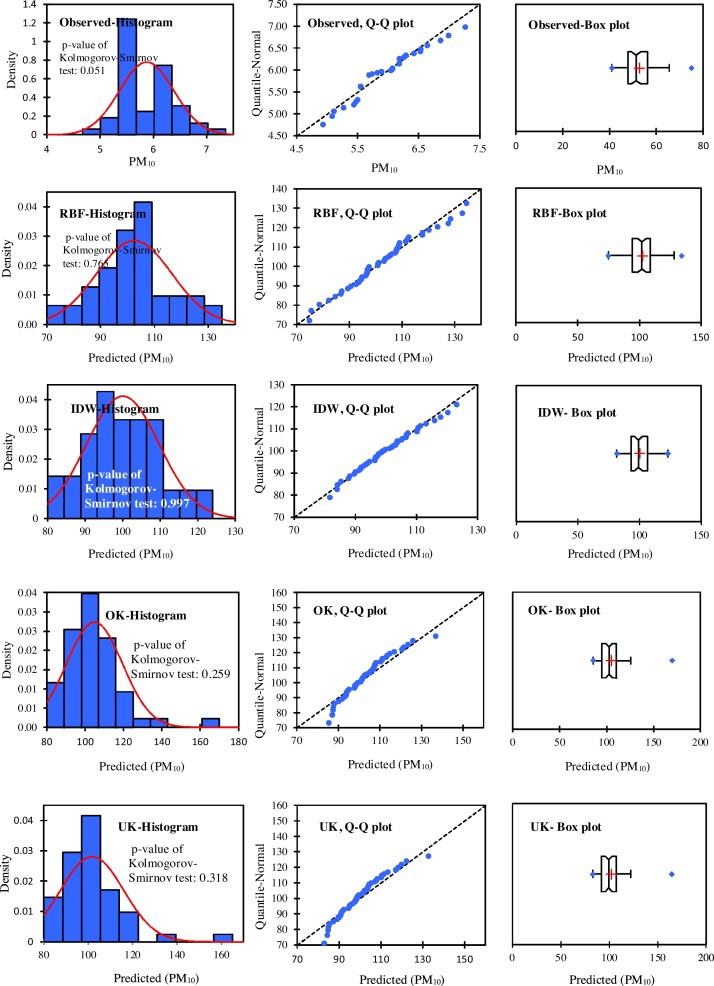
Comparison among the distributions of observed data, the predicted data using IDW, RBF, OK (stable function), and UK (stable function) for PM_10_. The figures were drawn by XLSTAT software.

In this study, the data from 48 stations were normalized by applying Box-Cox method and the coefficient of 0.6 for PM_2.5_ and 0.1 for PM_10_. The Box-Cox transformation is a particularly useful family of transformations. Normality assumptions are critical for many univariate intervals and hypothesis testing. It is important to test the normality assumption. If the data are in fact not normal, the Box-Cox normality plot can often be used to find a transformation that will approximately normalize the data. The distributions and normality of the observed data and the predicted values using the RBF, IDW, OK, and UK techniques were analyzed by using the histograms, the Q–Q plots, and the box plots (Fig. 4, Fig. 5). According to the results of this research, all points were fitted along with a straight line. Thus, this is the indication of normality of measured data (Fig. 4, Fig. 5: observed, Q–Q plot). Fig. 4, Fig. 5 show different distributions. Fig. 4, RBF histogram shows an example close to a normal distribution; OK and UK histograms show a distribution slightly right skewed; while IDW histogram have normal distribution (*p*-value of Kolmogorov-Smirnov test = 1.000). Fig. 5 shows that the *p*-value of Kolmogorov-Smirnov test for IDW is 0.997 and OK box plot shows a distribution highly skewed to the right. These results indicate that the performance of IDW is slightly better than the other methods.

**Fig. 6:**
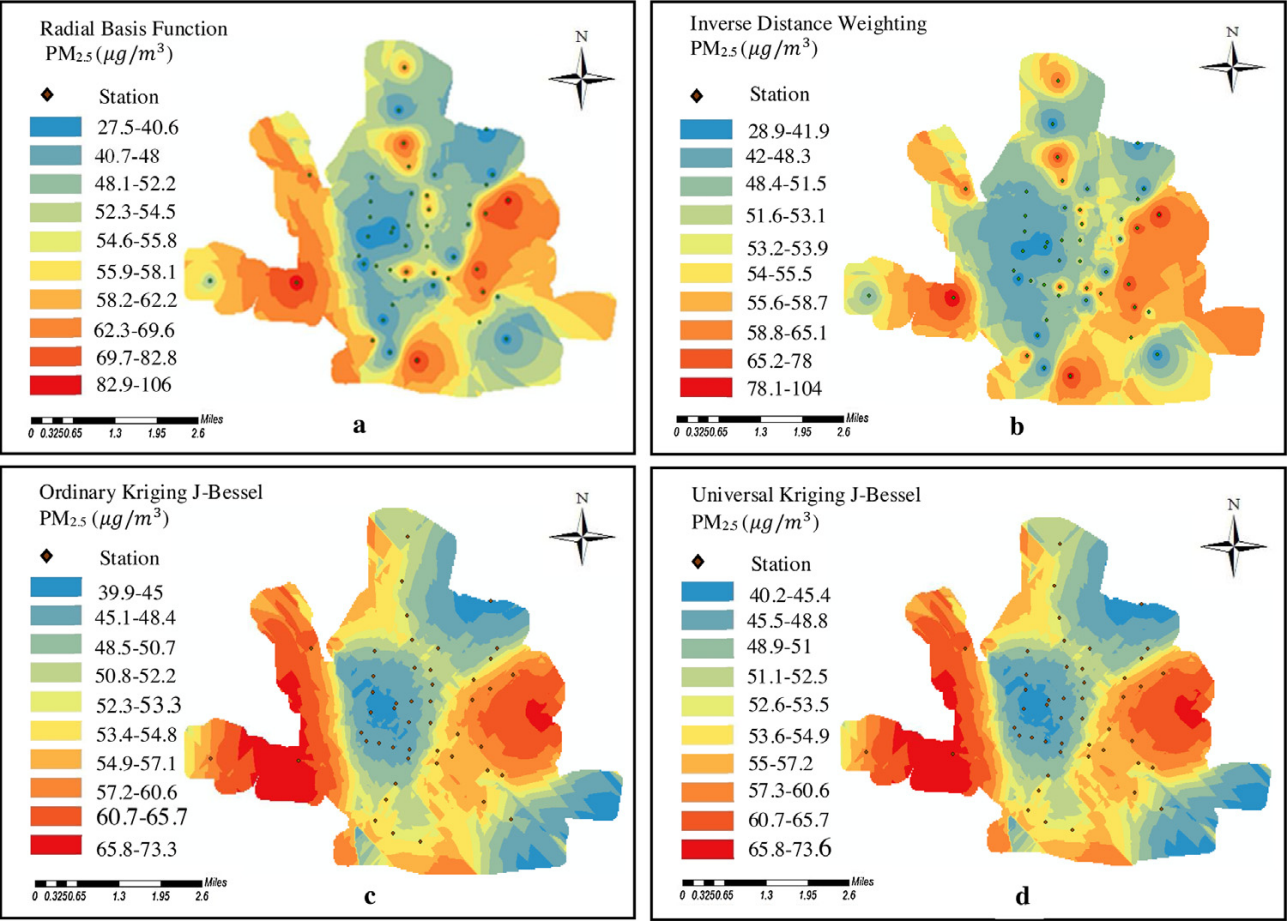
(a) Map of PM_2.5_ mean concentrations during 9-month study period estimated by RBF, (b) Map of PM_2.5_ concentrations estimated by IDW, (c) Map of PM_2.5_ concentrations estimated by OK, and (d) Map of PM_2.5_ concentrations estimated by UK.

**Fig. 7:**
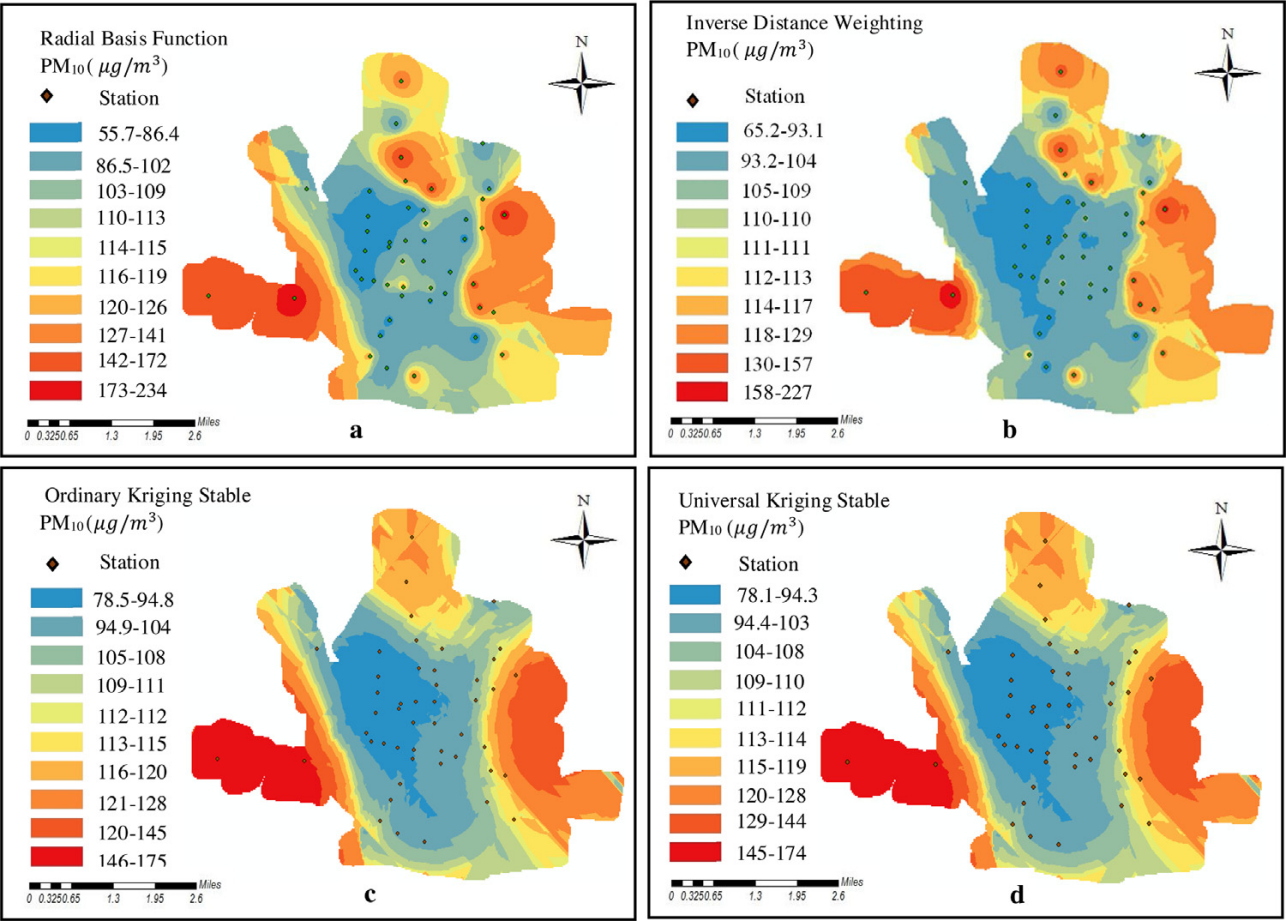
(a) Map of PM_10_ mean concentrations during 9-month study period estimated by RBF, (b) Map of PM_10_ concentrations estimated by IDW, (c) Map of PM_10_ concentrations estimated by OK, and (d) Map of PM_10_ concentrations estimated by UK.

**Fig. 8:**
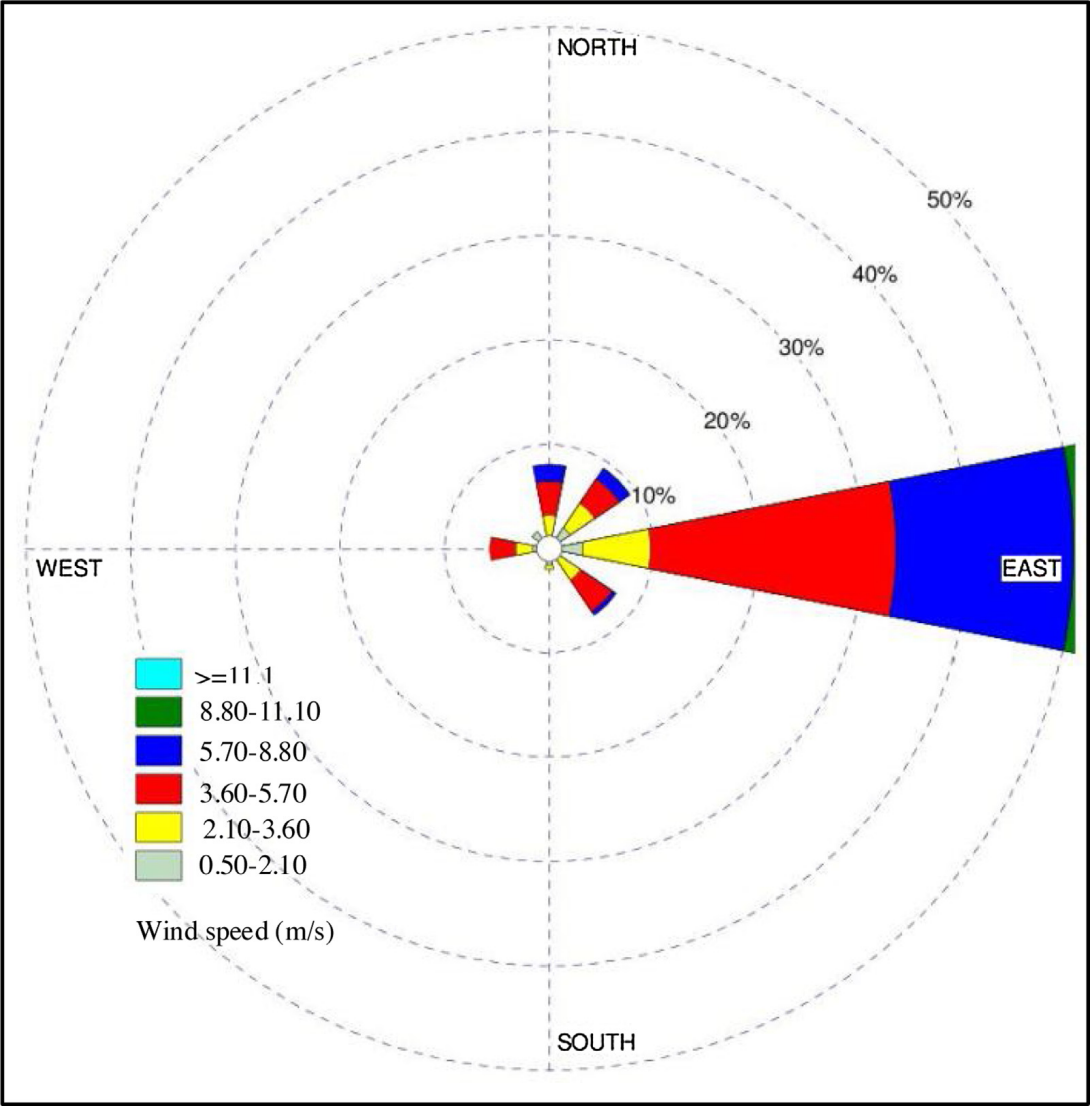
Wind rose of Sabzevar during April 2016–December 2016 (for nine month).

It is worth mentioning that in the present study the mean concentration during the 9-month study period were used in order to draw the distribution map. After applying interpolation function based on the above-mentioned method, pollution zoning for each pollutant (PM_2.5_ and PM_10_ particulate matters) was prepared by using deterministic methods (Radial Basis Functions and Inverse Distance Weighting) and geostatistical methods (Ordinary Kriging and Universal Kriging) (Fig. 6, Fig. 7). One of the considerations in the location of the monitoring stations is the winds which transport particulate matters to the monitoring stations. Local meteorology data were used to investigate the wind pattern. The wind rose was plotted during the 9-month study period (Fig. 8). Eastern winds are prevalent during study period. In the official meteorological reports, long term records also show that the prevailing wind direction has been east, owing to the topography of the region. Looking at the maps of these methods (Fig. 6, Fig. 7), the pollution of particulate matters is observed in the eastern regions of Sabzevar, which may be due to the prevailing wind blowing from the east in Sabzevar. The pollution is also observed in the southwest caused by traffic could be the result of high traffic of heavy vehicles in the highway located in this region (Mashhad highway), which is the entrance to Sabzevar. Fig. 6, Fig. 7 show the generated maps of the PM_2.5_ and PM_10_ predicted using RBF, IDW, OK and UK for the data sets in Sabzevar. The Figures show a difference between predicted maps using the deterministic (RBF and IDW) and geostatistical methods (OK and UK) techniques. Fig. 6a and b shows more red zones than c and d. Fig. 6b shows how the IDW algorithm behaves when a high PM outlier is next to a low PM value; the abrupt change in prediction surface is not seen with other methods. From Fig. 6b, the IDW outperformed in estimating values from the observed data. Fig. 7 is the same way. In this (Fig. 6, Fig. 7), the contribution of sampling density is very significant. The large sampling density makes the performance of OK and UK worse than the performance of IDW and RBF. However, it is not enough to evaluate the model performance only through the estimated values and maps. Comparing the estimated values with the observed values was used to evaluate the performance of all IDW, RBF, OK and UK techniques in terms of the accuracy of estimates [24]. In this study, the comparison of performance between the interpolation techniques was achieved by using RMSE and MAPE. The lower value of MAPE and RMSE for each interpolation method indicates the optimality of that method. Generally, lower the estimated error, the interpolation method previously mentioned to prepare the maps of pollution zoning are more appropriate. Table 2 presents the comparison of MAPE and RMSE values for each method of PM_2.5_ and PM_10_ particulate matters. Moreover, two parameters of MBE and MAE were used. The MAE and the RMSE were used to evaluate the accuracy of predictions. The MAE is similar to the RMSE but less sensitive to large forecast errors. The RMSE is the square root of the variance of the residuals. It indicates the absolute fit of model to the data and how close the observed data points are to the model’s predicted values [25]. Whereas R-squared is a relative measure of fit, RMSE is an absolute measure of fit. As the square root of a variance, RMSE can be interpreted as the standard deviation of the unexplained variance, and has the useful property of belonging to the same units as the response variable. Lower values of RMSE indicate a better fit. As Table 2 shows, the value of RSME for IDW method is 0.023 (PM_2.5_) and 0.03733 (PM_10_), respectively which is the lowest value among the methods. Examining MBE revealed some points. First, the model estimates the value of the variable more or less. Second, it determined the value quantity. When MBE is equal to zero, it indicates that the model has estimated the value under investigation in a good way without any diversion. The accuracy of the model is determined by MAE parameter. Regarding MAE, the value of zero is the indication of 100% accuracy of the model, and more distance from zero reflects lower accuracy of the model. This method of investigation was applied for all models of zoning. In general, the results shown in Table 2 indicate some differences in the values of MAPE, RMSE, MBE, and MAE. This means IDW was better than other methods in estimating the values from observed data.

**Table 2:** Comparison of the interpolation accuracies achieved using MAPE, RMSE, MBE, and MAE.

Particulate matters	Evaluation methods	Models	Functions	MAPE	RMSE	MBE	MAE
PM_2.5_	Deterministic methods	RBF	Completely regularized spline	42.55	0.024	−0.00018	0.01927
		IDW	Power 1	41.05	0.023	−0.00013	0.01870
			Power 2	42.09	0.024	−0.00020	0.01940
	Geostatistics methods	OK	Stable	44.16	0.024	−0.0017	0.01954
			Hole effect	44.22	0.025	−0.0013	0.01951
			J-Bessel	44.10	0.024	−0.0025	0.01956
			Guassian	44.16	0.024	−0.0017	0.01954
		UK	Stable	43.80	0.024	−0.0072	0.01954
			Hole effect	43.85	0.025	−0.0068	0.01958
			J-Bessel	43.74	0.025	−0.0080	0.01955
			Guassian	43.80	0.024	−0.0070	0.01954
PM_10_	Deterministic methods	RBF	Completely Regularized Spline	26.41	0.03865	−0.0019	0.02810
		IDW	Power 1	25.89	0.03733	−0.0017	0.02846
			Power 2	26.51	0.03886	−0.0041	0.02889
	Geostatistics methods	OK	Stable	27.19	0.03818	−0.0054	0.02838
			Hole Effect	27.54	0.03852	−0.0065	0.02880
			J-Bessel	27.43	0.03841	−0.0061	0.02865
			Guassian	27.26	0.03816	−0.002	0.02844
		UK	Stable	35.45	0.03843	0.0143	0.02794
			Hole Effect	36.06	0.03874	0.0147	0.02834
			J-Bessel	35.95	0.03864	0.0147	0.02820
			Guassian	35.64	0.03841	0.0145	0.02798

Karydas et al. [4] showed that the applied interpolation methods have similar results in terms of accuracy without clear advantages than to each other. Furthermore, in another study [26], no differences were found between Kriging and IDW methods for some elements, and MBA, MAE and RMSE factors were similar for these two methods; but for other studied elements, Kriging estimator showed a higher accuracy. Halek el al. [17] estimated urban suspended particulate in Tehran. The model was made based on the data from 42 stations located within the region. To build a “Surface Model” for PM_10_, PM_2.5_ and PM_1.0_, different algorithms were used to interpolate the data from those obtained for the known site. Then, the results were extended to the “surface”. For this purpose, mean concentrations of PM_10_, PM_2.5_ and PM_1.0_, in each sampling site for June, July and August were calculated, interpolated and generalized to the surface by “Inverse Distance Weight” or “Spine” algorithms by using ArcGIS 9.2. The results strongly indicated that the concentrations of PM_10_, PM_2.5_ and PM_1.0_ of any points inside the region, including the traffic zone, fail to meet the required international standard values. The extracted estimate concentrations for the 22 hospitals reveal that the concentration of PM_10_ for “Azadi Psychic”, “Children” and “Mustafa Khomeini” hospitals are the worst, estimating from the model to be 119.42 μg/m^3^, 107.09 μg/m^3^ and 101.14 μg/m^3^ respectively. Understanding spatial variability of air pollutant concentrations appear to be critical for public health assessments. Bermana et al. [27] examined ground-level ozone and evaluated the performance method for predicting and mapping national concentrations across the United States, while assessing the significance of accounting for spatial variability. Ozone concentration was predicted by using four approaches, including Land Use Regression (LUR), IDW, OK, and UK, and evaluated with a Monte Carlo cross-validation simulation. Results were mapped for the continental United States. UK was preferred over OK by allowing us to assess the significance of environmental covariates both for inference and prediction of ozone concentrations. In another research [28], two different methods, namely as Kriging method and Inverse Distance Weighted method, were examined for developing Digital Elevation Model image. Each method’s advantages and disadvantages were considered. Here, the Kriging yielded better estimates.

### Multiple regressions of particulate matters

A multiple regressions was calculated between particulate matter and independent meteorological parameters. Furthermore, the Spearman correlation test was used to examine the relationship between particulate matter and meteorological parameters. All the results indicated a significant relationship. The relationship of PM_2.5_ and meteorological parameters was positive for temperature (*p *= 0.046 and Pearson coefficient = 0.25) and wind speed (*p *= 0.006 and Pearson coefficient = 0.60) and negative for relative humidity (*p *= 0.001 and Pearson coefficient = −0.223) and precipitation (*p *= 0.03 and Pearson coefficient = −0.10). Furthermore, the relationship of PM_10_ and meteorological parameters was positive for temperature (*p *= 0.046 and Pearson coefficient = 0.23) and wind speed (*p *= 0.006 and Pearson coefficient=0.58) and negative for relative humidity (*p *= 0.001 and Pearson coefficient = −0.221) and precipitation (*p *= 0.03 and Pearson coefficient = −0.11). Considering the significant relationship between meteorological parameters and the pollutants, meteorological variables were found to be effective in the air pollution of Sabzevar. The results of this study revealed that the highest concentration of particulate matter has occurred at high temperatures. In other words, with increased temperature, the concentration has increased as well, since the correlation between particulates and temperature is positive and significant. According to the results of statistical surveys, the maximum concentrations of pollutants have occurred in the low relative humidity rate. In other words, increased air dryness has been associated with an increase in the amounts of pollutants. Increased relative humidity, if accompanied by a rainfall phenomenon, can reduce the air pollutants through washing. Therefore, this is one of the main factors in reducing the amounts of pollutants during high relative humidity and rainfall. There is a positive correlation between the mean concentration of particulate matter and the wind speed. The results of this study are similar to the results of the study entitled as “The relationship between urban air pollution and meteorological data in 2005 in the city of Cairo, Egypt” [29]. The Spearman correlation test was used to examine the relationship between PM _2.5_ and PM_10_ particulates. The variations of 24 h simultaneous PM_10_ and PM_2.5_ concentrations were correlated with R = 0.76 (*p *= 0.000). This finding showed the strong correlation between PM_10_ and PM_2.5_ concentrations.

## Additional information

Environmental monitoring can be defined as the systematic sampling of air, water, soil, and biota in order to observe and study the environment, as well as to derive knowledge from this process. Monitoring can be conducted for a number of purposes, including use of data to control the environment [30], [31], [32], to establish environmental “baselines, trends, and cumulative effects”, to test environmental modeling processes, to educate the public about environmental conditions, to inform policy design and decision-making, to ensure compliance with environmental regulations, to assess the effects of anthropogenic influences, or to conduct an inventory of natural resources [33], [34], [35]. Air pollution appears to be one of major dilemmas of urbanization and crowded cities. Particulate matter from the perspective of public health seems to be a major air pollutant. Concerns over ambient fine particulate matter (PM) pollution are increasing in the modern world due to its potentially harmful effects on the human health and the environment [36]. Dust may lead to climate changes on a global scale and local, as well as inducing changes in biological cycles and the environment. For, air quality is a major factor in the public health, which depends on the concentrations of particulate matter. This has been confirmed by comparing PM concentrations with life expectancy [37]. PM_1.0_, PM_2.5_ and PM_10_ are particulate matter with an aerodynamic diameter less than 1.0, 2.5 and 10 μm, respectively. Particles smaller than 10 mμ (PM_10_), as a life expectancy index, include small liquid droplets and solid particles that can easily be inhaled deeply [38]. Once threshold PM concentration levels exceed over prolonged periods, particulate matter might can a wide range of disturbances and illnesses [39]. Inflammation of the eyes, lungs, and the skin are other adverse effects of personal PM exposure [40]. Due to harms to the health of people caused by exposures to air pollutants in urban areas, monitoring and forecasting of air quality parameters have become popular as an important topic in atmospheric and environmental research today [41].

In air pollution studies, the air quality models are used to predict and estimate the concentrations of one or more species within the space and time relevant to the dependent variables. Modeling enables us to assess the current as well as the future air quality to make “informed” policy decisions [42], [43], [44], [45]. Interpolation methods have been well developed to estimate values at unknown locations based upon values spatially sampled in GIS. They are characterized as either deterministic or stochastic depending on using statistical properties [46]. Deterministic models include Inverse Distance Weighted (IDW), Rectangular, Natural Neighbours, and Spline methods. The IDW interpolator assumes that each input point has a local influence, which diminishes with distance. It gives more weight to the points closer to the processing cell than those are in further distant. Geostatistics is the second method used, consisting of Kriging in its various forms, Ordinary Kriging (OK), Simple Kriging (SK), and Universal Kriging (UK) [47], [48]. Kriging is a geostatistical technique to estimate the values of random fields at unobserved points resulted from observing values at known locations. IDW and Kriging techniques are widely used interpolation techniques [4]. Kravchenko and Bullock [46] evaluated the effect of data variability and the strength of spatial correlation in the data on the performance of the grid soil sampling of different sampling density and two interpolation procedures, Ordinary Point Kriging and Optimal Inverse Distance Weighting. Wollenhaupt et al. [49] compared these two interpolation methods and concluded that the IDW is more accurate for zooning P and K levels of soil. Setianto and Triandini [28] assessed the Kriging interpolation method to be more accurate compared to the IDW. Berman et al. [27] evaluated many methods for spatial mapping and came to the conclusion that Kriging was more accurate for zoning O_3_ levels of air ambient. Summarizing the above, the accuracy of each method depends on the assumptions and subjective judgments made, such as using or not-using the results smoothness and linearity of interpolation functions. In a study, the concentrations of PM_10_, PM_2.5_ and PM_1.0_ were measured in urban areas of Tehran at warm and cold seasons, which data were applied in the related modeling using the Arc-GIS. To this end, the samples were collected from 42 sites in an 18 km^2^ region located in the west and central parts of Tehran. The mean concentrations of PM_1.0_, PM_2.5_ and PM_10_ were respectively reported as 13.14 μg/m^3^, 22.67 μg/m^3^ and 95.72 μg/m^3^ in the warm season; and 50.12 μg/m^3^, 70.72 μg/m^3^ and 193.86 μg/m3 in the cold season [50]. Shad et al. [51] measured the particulate matter with a mass median aerodynamic diameter of less than 10 μm with concentrations at 52 sample stations in Tehran to identify dangerous areas for the human health. Followed by PM_10_ data prediction, their results demonstrated that genetic algorithms can reduce the estimated error (3.74) compared to linear functions (8.94 and 12.29). In recent years, atmospheric models, such as GIS, have been used for environmental analysis and the related management for supporting the environmental decision makers in different countries. In this study, concentrations of PM_2.5_ and PM_10_ were found in urban areas of Sabzevar for the first time and the data were applied in the related modeling by using Arc-GIS. This study was an attempt to evaluate spatial interpolation methods to determine the concentration of PM_2.5_ and PM_10_ in Sabzevar and select the most suitable interpolation method for preparation of zoning maps of the particulate matter in GIS. Multiple regressions analysis were used to investigate the effect of independent variables (meteorological parameters) on particulate matters concentrations.

In this research, the mean concentrations for PM_2.5_ and PM_10_ were as 56.61 μg/m^3^ and 110.93 μg/m^3^, respectively. According to the results of this study, the high pollution of particulate matters is observed in the eastern regions of Sabzevar which may be due to the prevailing wind blowing from the east in Sabzevar. The high pollution is also observed in the southwest caused by traffic could be the result of high traffic of heavy vehicles in this highway, as an entrance to Sabzevar. The results from this study showed that concentrations of PM_2.5_ and PM_10_ are higher than the regulatory standards in most stations; this calls for urgent need for continuous monitoring and control of source point emission as well as proper awareness for the citizenry for health hazards associated with air pollution. The data distribution and the autocorrelation were set as parameters to evaluate RBF, IDW, OK, and UK techniques. The best interpolation method for the particulate matter (PM_2.5_ and PM_10_) was deterministic method by IDW function. In summary, one can say that the model is useful for the estimation of pollutant concentrations within urban areas.
